# Quantitative tissue characterization of cardiac myxomas by CMR-Mapping techniques validated by histology

**DOI:** 10.1186/1532-429X-18-S1-P110

**Published:** 2016-01-27

**Authors:** Sarah B Nasser, Adelina Doltra, Bernhard Schnackenburg, Katharina Wassilew, Daniel Messroghli, Tamar Bigvava, Burkert Pieske, Volkmar Falk, Rolf Gebker, Sebastian Kelle

**Affiliations:** 1 Cardiology Department, Dar Al Fouad Hospital, Cairo, Egypt; 2 grid.418209.60000000100000404Cardiology Department, German Heart Institute Berlin, Berlin, Germany; 3 Clinical Science, Philips Healthcare, Hamburg, Germany; 4 grid.418209.60000000100000404Cardiac Pathology Department, German Heart Institute Berlin, Berlin, Germany; 5 Cardiology Department, Tbilisi Heart and Vascular Clinic, Tbilisi, Georgia; 6 grid.418209.60000000100000404Cardiothoracic Surgery Department, German Heart Institute, Berlin, Germany

## Background

Non-invasive imaging modalities are crucial for the detection and diagnosis of cardiac myxomas. Newer cardiac magnetic resonance (CMR) techniques to assess, extra-cellular volume fraction (ECV) and T2 mapping provide quantitative evaluation of cardiac tissue. Our aim was to test their diagnostic value in the assessment of cardiac myxomas.

## Methods

10 patients with morphologically suspected cardiac myxomas on echocardiography and confirmed by post-operative histopathology were prospectively included. CMR was performed at 1.5 Tesla in all patients pre-operatively. Standard protocol for cardiac mass assessment included T1 mapping (pre and post contrast to calculate ECV) and T2 mapping. All data are reported as mean ± standard deviation.

## Results

Cardiac myxomas demonstrated significantly higher native T1 values than the myocardium with a mean of 1489 ms ± 270 and 1024 ms ± 131 respectively (p = 0.007). However, cardiac myxomas showed a non-significant trend to lower mean post contrast T1 value than the myocardium, 406 ms ± 81 and 444 ms ± 23 respectively (p = 0.24) (see figure). The mean ECV for cardiac myxomas and myocardium was 45% ± 14% and 31% ± 5% respectively (p = 0.013). Mean T2 values for cardiac myxomas were also significantly higher than for the myocardium 154 ms ± 32 and 58 ms ± 4 respectively (p = 0.028).

## Conclusions

Compared to myocardium, we observed significantly higher native T1 values and increased ECV reflecting the different tissue composition and a larger extracellular interstitial compartment in cardiac myxomas. In addition, high T2 mapping values may indicate a higher fluid content in cardiac myxomas. CMR-mapping techniques might help to quantitatively assess cardiac myxomas non-invasively.

**Figure 1:**
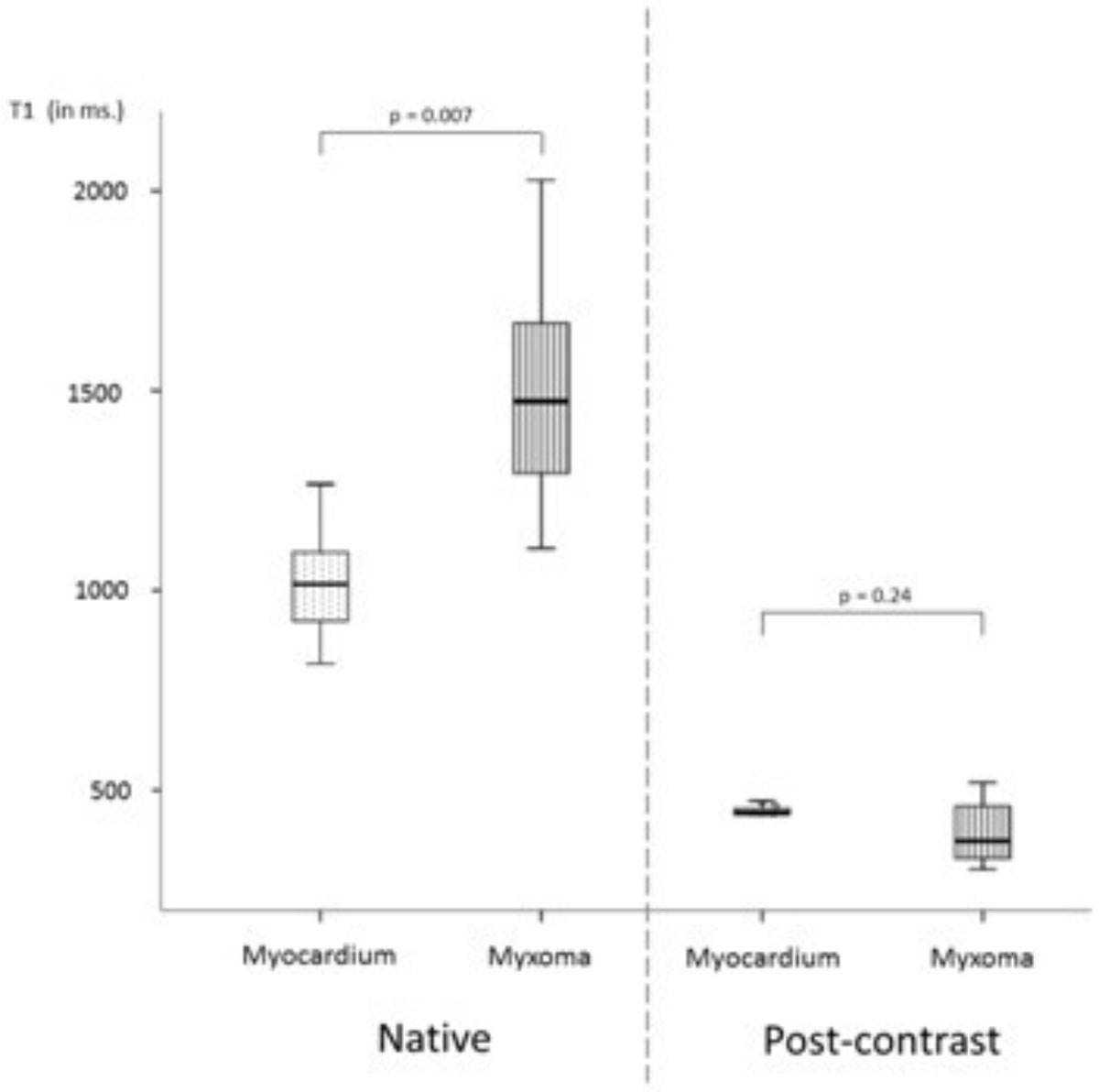
**T1 mapping values (in milliseconds) before (native) and post contrast administration for myocardium and cardiac myxomas**.

